# Successful use of ixekizumab for glucocorticoid-free remission maintenance in giant cell arteritis

**DOI:** 10.1093/rheumatology/keac416

**Published:** 2022-07-21

**Authors:** Alessandro Tomelleri, Emma Rinaldi, Corrado Campochiaro, Maria Picchio, Lorenzo Dagna

**Affiliations:** Vita-Salute San Raffaele University; Unit of Immunology, Rheumatology, Allergy and Rare Diseases; Vita-Salute San Raffaele University; Vita-Salute San Raffaele University; Unit of Immunology, Rheumatology, Allergy and Rare Diseases; Vita-Salute San Raffaele University; Nuclear Medicine Department, IRCCS San Raffaele Hospital, Milan, Italy; Vita-Salute San Raffaele University; Unit of Immunology, Rheumatology, Allergy and Rare Diseases

Rheumatology key message

- IL-17 blockade with ixekizumab can be a promising steroid-sparing agent in giant cell arteritis.

Dear Editor, GCA is a systemic vasculitis that primarily involves large- and medium-size arteries [1]. Glucocorticoids represent the cornerstone of GCA treatment, but their use is associated with significant toxicity [2]. Moreover, patients treated with glucocorticoid monotherapy experience a high relapse rate upon dose reduction or discontinuation [2]. The IL-6 receptor antagonist tocilizumab is the only approved treatment with confirmed efficacy in terms of remission maintenance and glucocorticoid-sparing effect in GCA [3]. However, tocilizumab makes CRP and ESR unreliable for monitoring disease activity [3]. Thus novel effective treatments are under evaluation. Herein we report for the first time the successful use of ixekizumab in maintaining clinical and radiologic remission in a GCA patient with concomitant skin psoriasis.

A 64-year-old man presented to our clinic in October 2019 with a 2-week history of right-sided headache and pelvic girdle pain and stiffness. He reported a 5-year history of skin psoriasis, successfully treated with methotrexate 7.5 mg weekly. His medical history was also significant for arterial hypertension and type 2 diabetes mellitus. On physical examination, the right temporal artery was tender and swollen. Laboratory tests showed elevated CRP (57 mg/l) and ESR (63 mm/h). A temporal artery ultrasound disclosed bilateral temporal and parietal non-compressible halo sign, in keeping with GCA. High-dose glucocorticoids (prednisone 50 mg/day) were started, while the methotrexate dose was maintained unaltered. Seven days after therapy start, a ^18^F-fluorodeoxyglucose PET (FDG-PET) showed significantly increased uptake (Meller score 3) in the left subclavian and common carotid artery and in both femoral arteries [PET vascular activity score (PETVAS) 6; see Fig. 1A, B] [4]. Glucocorticoid therapy led to a rapid resolution of symptoms and to a normalization of inflammatory markers. Two months later, while on prednisone 20 mg/day, the methotrexate dose was increased to 15 mg weekly due to a slight increase in inflammatory markers (CRP 9.6 mg/l, ESR 35 mm/h). However, 7 months after therapy start, inflammatory markers were still above the normal limits (CRP 11.3 mg/l, ESR 63 mm/h) and the patient experienced a polymyalgic flare. Tocilizumab 162 mg weekly was introduced with satisfactory clinical control, thus allowing glucocorticoids withdrawal. A repeated FDG-PET showed persistence of low metabolic activity over the bilateral common carotid and subclavian arteries (PETVAS 6). Nevertheless, therapy was not modified. In January 2021, the patient had an exacerbation of skin psoriasis on the scalp, elbows and hands. A course of topical steroid therapy was given, but was ineffective. Therefore, as suggested by the caring dermatologist, we agreed on switching treatment from tocilizumab to ixekizumab (160 mg followed by 80 mg every 4 weeks). Ixekizumab was extremely effective on the skin psoriasis, which disappeared after only 2 months. In addition, despite tocilizumab suspension, in the following 12 months the patient did not experience any recurrence of GCA symptoms and inflammatory markers remained within normal limits. A repeated FDG-PET scan performed in March 2022 (1 year after ixekizumab start) showed no evidence of residual large-vessel vasculitis (PETVAS 1) (Fig. 1C, D).

**Fig. 1:**
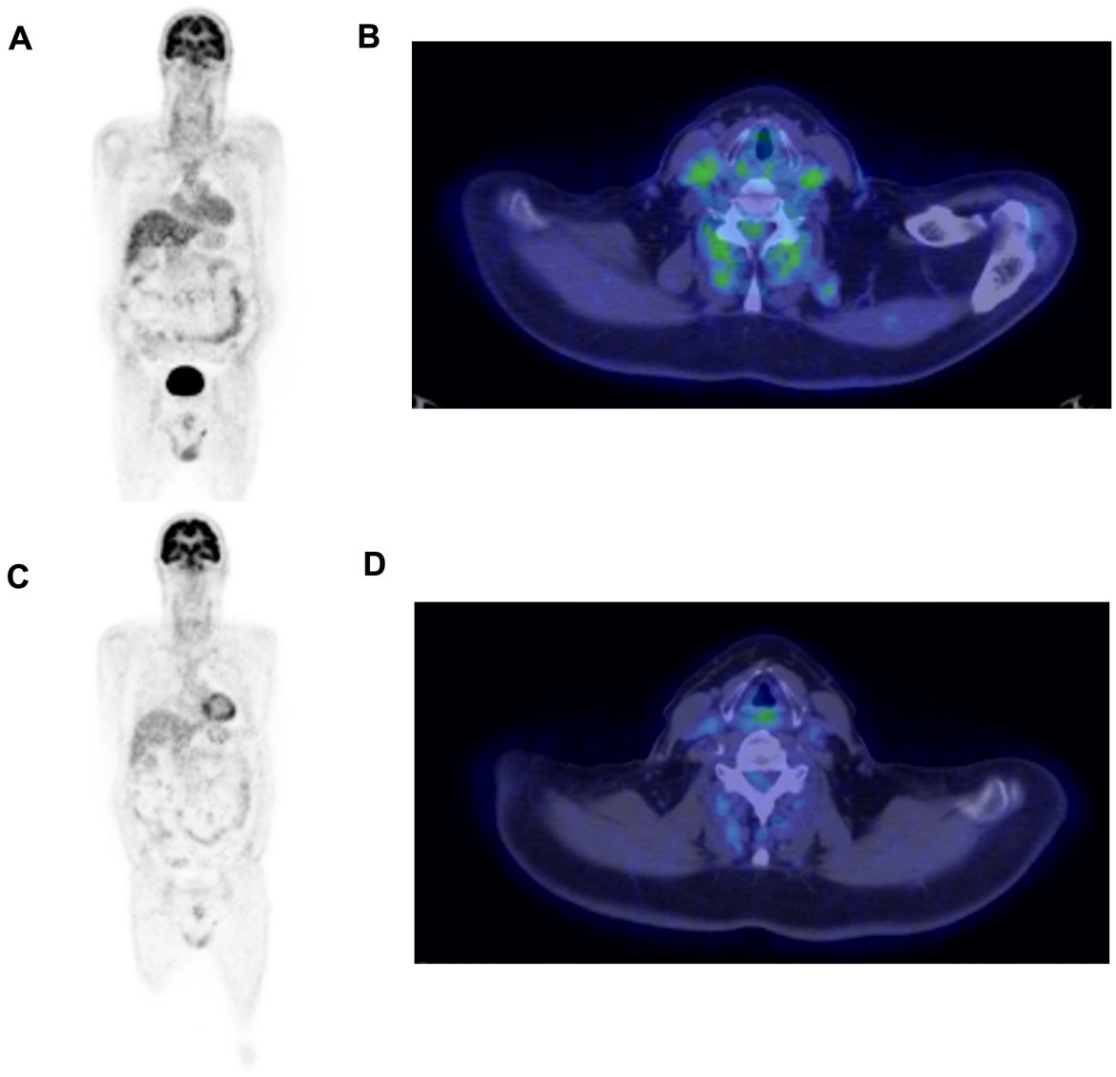
^18^F-FDG PET/CT scan at disease onset and after 3 years **(A, B)** ^18^F-FDG PET/CT performed at disease onset shows pathological radiotracer uptake by the left subclavian artery, the proximal tract of the left brachial artery and the left common carotid artery. **(C, D)**^18^F-FDG PET/CT performed 3 years later (1 year after ixekizumab start) shows complete resolution of these original findings.

IL-17 is thought to exert a significant role in GCA pathogenesis. It is produced by Th-17 cells infiltrating GCA lesions and is responsible for pleiotropic effects on a variety of cells, promoting vascular and systemic inflammation [5]. Rotar *et al.* [6] described the first case of GCA remission maintenance achieved with secukinumab, a monoclonal anti-IL-17A antibody, in a patient with concurrent psoriatic arthritis. This first observation was followed by the positive results of a phase 2 placebo-controlled trial evaluating the efficacy and safety of IL-17 inhibition with secukinumab in newly diagnosed or relapsing GCA patients. In this trial, the proportion of patients in sustained remission at week 28 was significantly higher in the treatment arm (70.1% *vs* 20.3%); in addition, in this group, time to first flare was longer [7]. A phase 3 study with a larger number of patients has recently started recruitment (NCT04930094). Ixekizumab is a monoclonal antibody targeting IL-17A with efficacy and safety profiles similar to secukinumab in the treatment of skin psoriasis and psoriatic arthritis [8]. However, its use in patients with GCA has never been previously investigated. As the results of the phase 2 study had not yet been published by the time we decided to start our patient on IL-17 inhibitor, we confirmed the choice of ixekizumab as proposed by his caring dermatologist.

Our case supports the hypothesis that interfering with the IL-17 signalling pathway might be a promising option for the maintenance of glucocorticoid-free remission in GCA and that ixekizumab might be a valid alternative to secukinumab in GCA. Replication of this preliminary observation in larger cohorts is required.

*Funding*: No specific funding was received from any bodies in the public, commercial or not-for-profit sectors to carry out the work described in this article.

*Disclosure statement*: The authors have declared no conflicts of interest. Informed consent was obtained from the patient.

## Data Availability

Data are available upon reasonable request by any qualified researchers who engage in rigorous, independent scientific research, and will be provided following review and approval of a research proposal and Statistical Analysis Plan (SAP) and execution of a Data Sharing Agreement (DSA). All data relevant to the study are included in the article.
